# Multisystem Langerhans cell Histiocytosis presenting with spontaneous pneumothorax in a toddler: case report and literature review

**DOI:** 10.1093/omcr/omaf255

**Published:** 2025-12-26

**Authors:** Subhash Chandra Tard, Ayushi Shrivastav, Gajanand S Tanwar, Mukesh Beniwal

**Affiliations:** Department of Pediatrics, Sardar Patel Medical College, Bikaner, SPMC Road, PBM Hospital Bikaner, Rajasthan 334001, India; Department of Pediatrics, Sardar Patel Medical College, Bikaner, SPMC Road, PBM Hospital Bikaner, Rajasthan 334001, India; Department of Pediatrics, Sardar Patel Medical College, Bikaner, SPMC Road, PBM Hospital Bikaner, Rajasthan 334001, India; Department of Pediatrics, Sardar Patel Medical College, Bikaner, SPMC Road, PBM Hospital Bikaner, Rajasthan 334001, India

**Keywords:** Langerhans cell histiocytosis, pulmonary histiocytosis, pneumothorax, cystic lung disease, pediatric interstitial lung disease, vinblastine therapy

## Abstract

Multisystem Langerhans cell histiocytosis (MS-LCH) is exceedingly rare in very young children and can present with predominant lung involvement. We report a 2-year-old boy who presented with prolonged fever, cough, and acute respiratory distress. Chest imaging revealed recurrent bilateral pneumothoraces requiring multiple chest drains; high-resolution computed tomography showed diffuse bilateral thin-walled lung cysts. Thoracoscopic lung biopsy with immunohistochemistry (CD1a+, CD45+, S100+) confirmed Langerhans cell histiocytosis. Given the strong association between pediatric pulmonary LCH and multisystem disease, a whole-body FDG-PET/CT was performed, revealing hepatic and splenic involvement and confirming a diagnosis of MS-LCH. Systemic chemotherapy with vinblastine and corticosteroids led to clinical improvement. We also review nine similar pediatric cases reported in the literature, highlighting the importance of recognizing pulmonary findings as a gateway to diagnosing underlying multisystem LCH.

## Introduction

Langerhans cell histiocytosis (LCH) is a clonal myeloid neoplasm of CD1a-positive dendritic cells, with an estimated pediatric incidence of ~ 5 per million children and a peak occurrence between 1 and 4 years of age [1]. LCH may present as a single-site lesion (e.g. eosinophilic granuloma) or as multisystem disease, and involvement of risk organs (liver, spleen, bone marrow) predicts a worse prognosis. Although bone, skin, and the pituitary gland are the most frequent sites, pulmonary lesions in children almost always occur in the context of multisystem disease rather than in isolation. In adults, pulmonary LCH is classically linked to smoking, but in pediatric patients, pulmonary involvement often mimics other interstitial lung diseases or infections, leading to diagnostic delay. Recurrent spontaneous pneumothorax is a particularly rare but recognized complication of pediatric multisystem LCH.

## Case presentation

**Figure 1:**
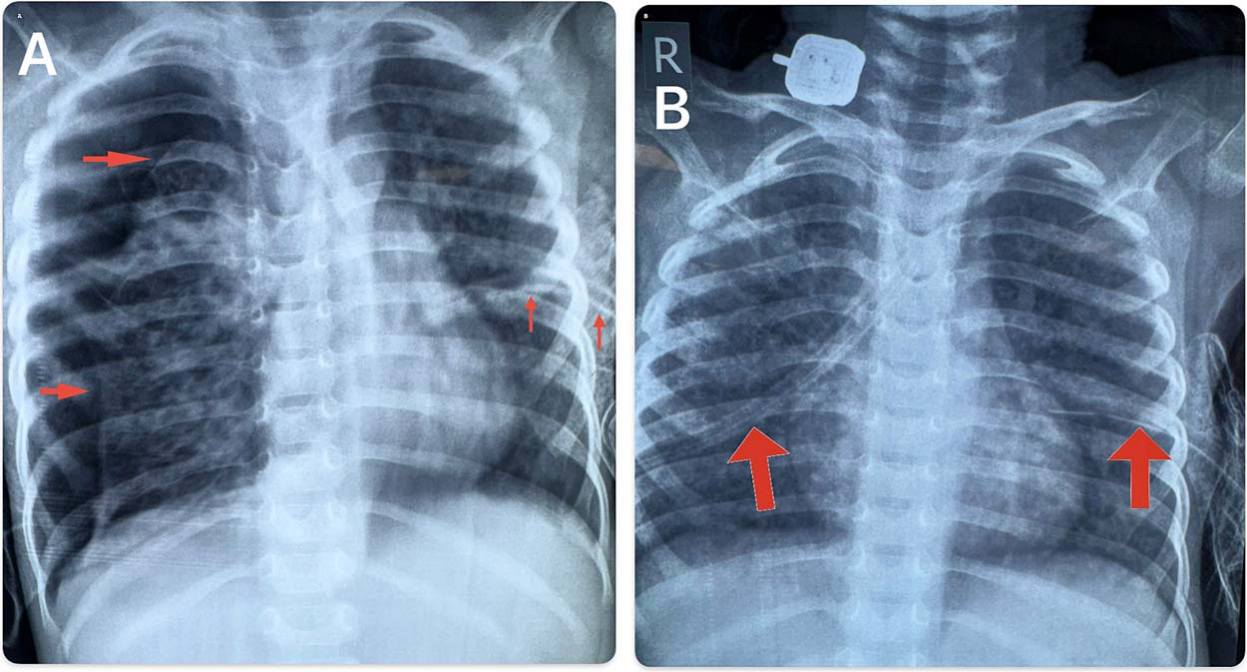
(A-B): Combined chest X-rays. (A) Chest X-ray showing a left-sided intercostal chest drain (ICD) in situ with a right-sided pneumothorax. (B) Chest X-ray after ICD insertion showing lung re-expansion with resolving pneumothorax.

A previously healthy 2-year-old boy presented with 20 days of high-grade fever and 10 days of cough. On the eve of admission, he developed acute respiratory distress. Initial chest radiography showed a large left pneumothorax, and a chest tube (intercostal drain) was placed. Over the next week, he had two further episodes of recurrent right pneumothorax requiring additional chest drains (Fig. 1A and B).

**Figure 2:**
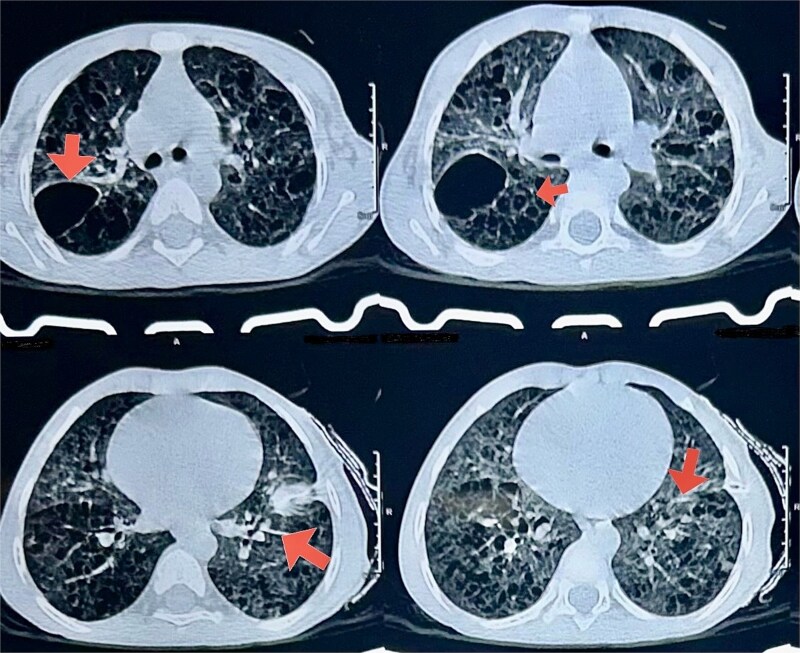
HRCT showing diffuse cystic lung disease.

The physical exam was notable only for tachypnea; there were no cutaneous lesions, lymphadenopathy, or organomegaly. Initial blood tests showed an elevated CRP (27.35 mg/L), a mildly elevated total leukocyte count (14 400/μL), and normal liver enzymes (SGOT: 26 U/L, SGPT: 31 U/L), and extended workup including CBNAAT, MT, ESR and AFB was negative, blood culture reports sterile, other relevant test normal as shown in (Table 1). In following days, child developed recurrent pneumothoraces, prompting a high-resolution CT (HRCT) of the chest, which revealed numerous bilateral thin-walled cysts and pneumatoceles, most prominent in the mid and upper lung zones (Fig. 2).

**Table 1:** Summary of laboratory parameters with reference ranges and interpretations

Parameter	Observed Value	Reference Range (Pediatrics)	Interpretation
Haemoglobin (gm/dL)	11.6	11.0–14.0	Normal
Total Leukocyte Count (10^3^/μL)	14.44	5.0–13.0	High
Platelet Count (10^3^/μL)	556	150–450	High
MCV (fL)	64.5	75–90	Low (microcytosis)
MCH (pg)	19.5	24–30	Low
Neutrophils (%)	33.2	37–75	Low
Hematocrit (%)	38.3	36.0–51.0	Normal
CRP (mg/L)	27.35	0.0–6.0	High (inflammation)
ESR (mm/hr)	7	10–15	Normal
Blood Culture	No growth in 72 hrs	—	Normal
Clotting Time (min)	6.25	3.0–8.0	Normal
HIV (Rapid)	Non-Reactive	Non-Reactive	Normal
HbsAg (Rapid)	Negative	Negative	Normal
Serum Sugar (Random)	90 mg/dL	70–110	Normal
Serum Sugar (Fasting)	87 mg/dL	70–110	Normal
Serum Urea (mg/dL)	17	10–45	Normal
Serum Creatinine (mg/dL)	0.6	0.6–1.6	Normal
Serum Bilirubin (Total)	0.2	Up to 1.2	Normal
Serum Bilirubin (Direct)	0.07	Up to 0.5	Normal
SGOT/AST (IU/L)	26	Up to 40	Normal
SGPT/ALT (IU/L)	31	Up to 35	Normal
Alkaline Phosphatase (IU/L)	240	210–810 (for child < 15 years)	Normal
Mantoux Test(PPD,mm)	No Induration (3rd day)	—	Negative
CBNAAT	Not Detected	*Mycobacterium tuberculosis* not detected	Normal

Findings were suggestive of diffuse cystic lung disease. Alternative diagnoses including congenital pulmonary airway malformation (CPAM), bronchogenic cyst, and post-infectious pneumatocele were considered but deemed unlikely based on the clinical picture and imaging characteristics. A video-assisted thoracoscopic lung biopsy was obtained. Histopathology demonstrated characteristic Langerhans cells (reniform nuclei, abundant cytoplasm) admixed with eosinophils. Immunohistochemistry was strongly positive for CD1a, CD45, and S100, confirming LCH [2].

**Figure 3:**
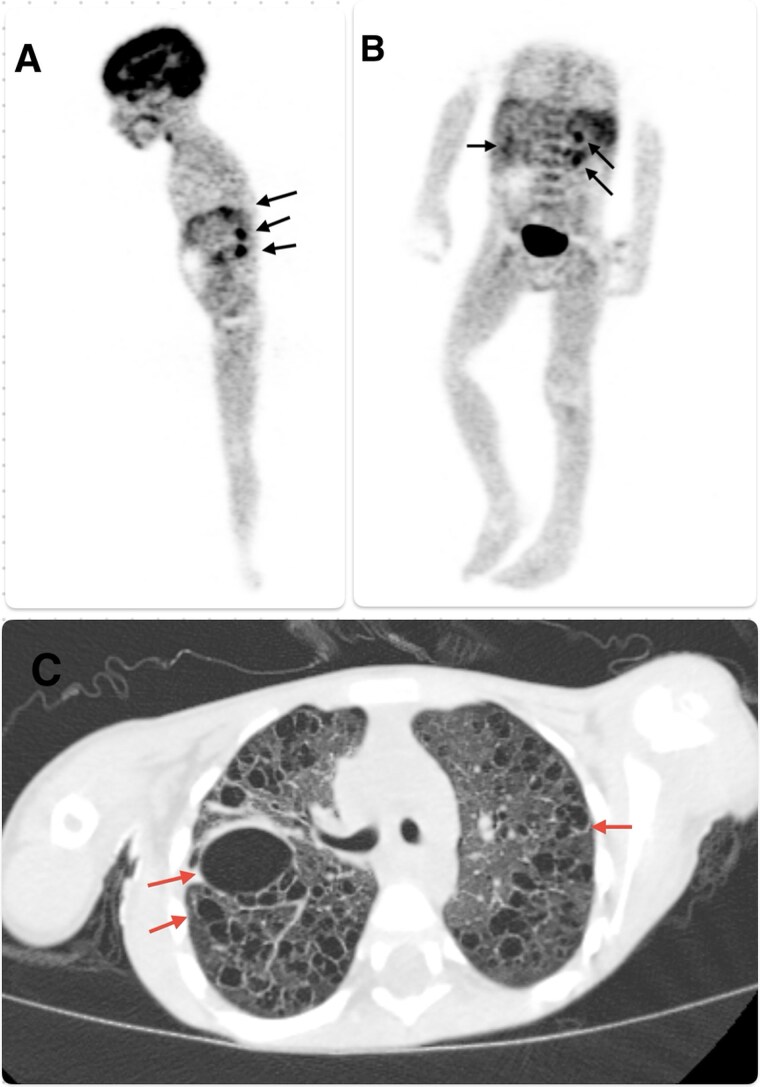
Whole-body FDG-PET/CT and axial chest CT in multisystem LCH. (A) Sagittal maximum-intensity-projection (MIP) FDG-PET image demonstrating mildly increased FDG uptake (black arrows) in extensive bilateral pulmonary cystic lesions. (B) Coronal MIP FDG-PET image showing FDG-avid pulmonary cysts (black arrows), mildly metabolically active subcentimetre to enlarged supra- and infradiaphragmatic lymphadenopathy, and hepatosplenomegaly with diffuse splenic hypermetabolism. (C) Axial high-resolution CT through the upper lobes depicting numerous thin-walled cysts of varying sizes in both lungs (red arrows), including a large right upper-lobe cyst (central arrow). Abbreviations: FDG, 2-deoxy-2-[^18F]fluoro-D-glucose; PET, positron-emission tomography; CT, computed tomography; MIP, maximum-intensity projection; LCH, Langerhans cell histiocytosis; HRCT, high-resolution CT.

Staging FDG-PET/CT identified metabolically active pulmonary lesions and showed hepatic and splenic involvement consistent with multisystem disease (Fig. 3). No bone lesions or diabetes insipidus were identified, Given multisystem LCH with risk-organ involvement (liver and spleen), the patient was started on systemic chemotherapy. He received weekly intravenous vinblastine and daily oral prednisolone during an initial 6-week induction phase, with a planned 12-month continuation.The child’s fevers resolved and respiratory status improved. The chest tubes were eventually removed without further pneumothorax. He was discharged in stable condition and continues on outpatient chemotherapy with improving clinical status.

## Discussion

Langerhans cell histiocytosis with pulmonary involvement is uncommon in children, accounting for roughly 7–16% of pediatric LCH cases. In one large pediatric series, only about 10% of children with LCH had lung involvement [3]. Most pediatric pulmonary LCH occurs as part of multisystem disease rather than as isolated lung disease. Our case exemplifies this pattern, the child’s pulmonary manifestations were part of a systemic process confirmed by imaging.

The etiology of LCH remains incompletely understood; it is now classified as a myeloid neoplastic disorder with recurrent activating mutations in the MAPK pathway. Notably, somatic BRAF V600E mutations are found in roughly half of LCH lesions. Other MAPK mutations (e.g. MAP2K1, ARAF) occur in additional cases. Pulmonary LCH often presents with nonspecific respiratory symptoms, in reported pediatric series, less than one-third of children with pulmonary LCH had cough or dyspnea at diagnosis, and recurrent pneumothorax was noted in only a minority, in our patient, by contrast, experienced multiple spontaneous pneumothoraces.

Radiographically, LCH of the lung evolves through characteristic stages, early disease produces centrilobular nodules (<1 cm) reflecting peri-bronchiolar granulomas. Over time, these nodules cavitate to form cysts. Initially the cysts are thick-walled, but with disease evolution they become thin-walled and can coalesce into bizarre shapes. In our patient’s high-resolution CT scans, we saw diffuse, thin-walled cysts of variable size, with otherwise preserved lung parenchyma and occasional ground-glass opacities suggesting active inflammation. Late-stage disease can progress to fibrosis and honeycombing [4].

Given the diffuse bilateral thin-walled cysts on HRCT, we considered several alternative diagnoses. Congenital lesions such as congenital pulmonary airway malformation (CPAM) and focal bronchogenic cysts were unlikely because these entities are typically focal or lobar rather than diffuse and bilateral. Post-infectious pneumatoceles may appear following severe bacterial pneumonia (e.g. *Staphylococcus aureus*), but the clinical course and negative microbiology made this less likely in our patient. Surfactant protein deficiencies and other childhood interstitial lung diseases usually present with ground-glass attenuation and interstitial involvement rather than the predominant cystic pattern seen here. Microbiological testing including CBNAAT, AFB smear, and blood cultures were negative. In summary, while these differentials were considered, the imaging distribution together with negative infectious workup made them unlikely, prompting PET staging, in our case PET uptake was noted in the pulmonary cystic lesions as well as in the liver and spleen, and there was mildly metabolically active supra- and infra-diaphragmatic lymphadenopathy. These findings confirmed risk-organ involvement and supported a diagnosis of multisystem LCH.

And for the definitive diagnosis of LCH requires biopsy which shows Characteristic Langerhans cells have reniform (‘coffee-bean’) nuclei and Birbeck granules (tennis-racket rods) on electron microscopy, Immunohistochemistry is diagnostic: lesional cells are positive for (CD1a) and Langerin (CD207) and variably (S100). Our patient’s lesion was strongly (CD1a) and (S100) positive.

We performed a PubMed-based literature review (May 2025) using combinations of the terms ‘LCH,’ ‘child,’ ‘preschool,’ ‘pulmonary,’ ‘pneumothorax,’ and ‘case report,’ identifying nine relevant pediatric cases of LCH with spontaneous pneumothorax. Of these, six cases from five studies were included in our analysis (Table 2) [5–9], based on data completeness and relevance. The remaining three, although similar in presentation, were excluded due to limited detail or duplication.

**Table 2:** Summary of previously reported pediatric cases of Langerhans cell histiocytosis (LCH) presenting with pneumothorax

No.	Author (Year)	Age/Sex	Imaging	Presentation	Treatment	Outcome (Follow-up)
1	Braier J et al. (2007) [5]	20 mo F	CXR/CT: bilateral cysts & bullae	Acute respiratory distress; bilateral PTX	Lung biopsy; prednisone + vinblastine + 6-MP; bilateral chest tubes	PTX resolved by ~ 5 years; asymptomatic; normal PFT
2	Martínez-García JJ et al. (2014) [6]	2 yr M	CT: cysts, fibrosis, bullae	Recurrent bilateral spontaneous PTX	Lung biopsy; prednisone + etoposide	Good response; improved CT
3	Verma S et al. (2014) [7]	2 yr M	CT: bilateral cystic lucencies	Fever, cough; developed PTX	Lung biopsy; iodopovidone pleurodesis	PTX resolved; no recurrence
4	Soyer OG et al. (2019) [8]	3 yr M	CXR: bilateral pneumothoraces	Severe distress; bilateral PTX; neck mass	Chest tube; vinblastine + prednisone; talc pleurodesis; thoracoscopy; ECMO	Died day 24 from ECMO complication
5	Carmo LHKD et al. (2024, Case 1) [9]	3 yr M	CXR: right pneumothorax	Right PTX with two contralateral recurrences	Bilateral VATS + thoracotomy; mechanical pleurodesis; chemotherapy	Good response; no further PTX
6	Carmo LHKD et al. (2024, Case 2)	4 yr M	CXR: right pneumothorax	Progressive dyspnea; right PTX	Chest tube; silver nitrate pleurodesis; chemotherapy	Good response; PTX resolved

Legend: PTX = pneumothorax; CXR = chest X-ray; CT = computed tomography; PFT = pulmonary function test; VATS = video-assisted thoracoscopic surgery; ECMO = extracorporeal membrane oxygenation.

Most reported patients were male preschoolers presenting with spontaneous pneumothorax and bilateral cystic lung changes on imaging. All required chest interventions such as thoracostomy, pleurodesis, or surgery alongside systemic chemotherapy, most commonly vinblastine and prednisone. Five of the six included cases had favorable outcomes with resolution of pneumothoraces. In contrast, a 3-year-old boy described by Soyer et al. [9] died despite aggressive interventions, including chemotherapy, pleurodesis, surgical resection, and ECMO.

Our patient, classified as high-risk multisystem LCH based on FDG-PET/CT evidence of liver and splenic involvement, had a similar presentation but responded well to vinblastine/prednisone and thoracostomy (inter costal drainage), without the need for surgical pleurodesis. He has remained recurrence-free on ongoing chemotherapy and supportive care [10].

## Conclusions

MS-LCH, although rare, should be considered in young children presenting with unexplained diffuse cystic lung disease and recurrent pneumothorax, as pulmonary involvement in this age group often indicates underlying systemic disease. Cystic lung lesions in toddlers can also delay diagnosis because they may initially be mistaken for congenital pulmonary airway malformation, bronchogenic cysts, or post-infectious pneumatoceles.

Prompt recognition of characteristic imaging patterns and early histopathological confirmation are essential for accurate diagnosis and early treatment of MS-LCH with pulmonary involvement can lead to remission. This case underscores that when LCH presents primarily with lung findings, a coordinated, multidisciplinary approach is essential.

## Declarations

Ethics approval and consent to participate: Not applicable (case reports do not require formal IRB approval at our institution).
